# Creating and Implementing a Community Engagement Strategy for the 2022–2027 Illinois Comprehensive Cancer Control Plan Through an Academic–State Public Health Department Partnership

**DOI:** 10.5888/pcd20.220422

**Published:** 2023-08-10

**Authors:** Leslie R. Carnahan, Colleen Hallock, Brenda Soto, Linda Kasebier, Elise Dracos, Erica Martinez, Jennifer Newsome, Tigist Mersha, David Pluta, Vida Henderson, Manorama Khare

**Affiliations:** 1 University of Illinois Cancer Center, Chicago, Illinois; 2 School of Public Health, University of Illinois Chicago, Chicago, Illinois; 3 Illinois Department of Public Health, Springfield, Illinois; 4 Foundation for the National Institutes of Health, North Bethesda, Maryland; 5 Department of Family and Community Medicine, University of Illinois College of Medicine Rockford, Rockford, Illinois; 6 Fred Hutchinson Cancer Research Center, Seattle, Washington

## Abstract

**Introduction:**

Comprehensive cancer control (CCC) plans are state-level blueprints that identify regional cancer priorities and health equity strategies. Coalitions are encouraged to engage with community members, advocacy groups, people representing multiple sectors, and working partners throughout the development process. We describe the community and legislative engagement strategy developed and implemented during 2020–2022 for the 2022–2027 Illinois CCC plan.

**Methods:**

The engagement strategies were grounded in theory and evidence-based tools and resources. It was developed and implemented by coalition members representing the state health department and an academic partner, with feedback from the larger coalition. The strategy included a statewide town hall, 8 focus groups, and raising awareness of the plan among state policy makers.

**Results:**

A total of 112 people participated in the town hall and focus groups, including 40 (36%) cancer survivors, 31 (28%) cancer caregivers, and 18 (16%) Latino and 26 (23%) African American residents. Fourteen of 53 (26%) focus group participants identified as rural. Participants identified drivers of cancer disparities (eg, lack of a comprehensive health insurance system, discrimination, transportation access) and funding and policy priorities. Illinois House Resolution 0675, the Illinois Cancer Control Plan, was passed in March 2022.

**Conclusion:**

The expertise and voices of community members affected by cancer can be documented and reflected in CCC plans. CCC plans can be brought to the attention of policy makers. Other coalitions working on state plans may consider replicating our strategy. Ultimately, CCC plans should reflect health equity principles and prioritize eliminating cancer disparities.

**Summary**

**What is already known on this topic?**

Comprehensive cancer control plans are important tools for guiding community and state-level activities that focus on cancer prevention and control by identifying priorities and health equity strategies to address the burden of cancer.

**What is added by this report?**

The development and implementation of these plans should include community members and people from multiple sectors.

**What are the implications for public health practice?**

This report provides a model of community engagement that can serve as a blueprint for other statewide cancer coalitions working on their own plans.

## Introduction

Comprehensive cancer control (CCC) plans are blueprints that identify region-specific cancer priorities and health equity strategies to address cancer prevention and control (1–3). The Centers for Disease Control and Prevention’s (CDC’s) National Comprehensive Cancer Control Program, established in 1998, supports CCC development and provides funding, guidance, and technical assistance to US territories and freely associated states, the District of Columbia, and tribes and tribal organizations, to design and implement plans (4). Plans guide cancer prevention and control activities with the goal of reducing cancer incidence and death rates by addressing all parts of the cancer continuum (1–3,5). Although including goals, objectives, and strategies is standard across plans, each plan is unique to its region, and content varies in scope, priorities, and length.

Statewide coalitions are responsible for creating CCC plans, and these plans generally span a 5-year period (3). When working on the development of CCC plans, coalitions are encouraged to engage people with diverse perspectives, such as community members, advocacy groups, people representing multiple sectors, and working partners (1,2,5–7). Meaningful community engagement can advance cancer health equity by informing practice, research, and policy with input from people who are typically marginalized and by identifying community-aligned solutions (8).

The Illinois Department of Public Health received funding from CDC to administer the Illinois Comprehensive Cancer Control Program and develop the 2022–2027 Illinois Comprehensive Cancer Control Plan in collaboration with its statewide coalition, the Illinois Cancer Partnership (ICP). The plan identifies how the state will address cancer with a focus on reducing cancer incidence and death rates through prevention, screening, early detection, and diagnosis, treatment, and survivorship, all with health equity–focused activities and strategies. A new addition to the 2022–2027 Illinois Comprehensive Cancer Control Plan is a robust, multipronged community engagement approach.

We describe the community engagement strategy developed and implemented for the 2022–2027 Illinois Comprehensive Cancer Control Plan. This model can serve as a blueprint for other statewide cancer coalitions working on their own CCC plans.

## Methods

**Figure 1:**
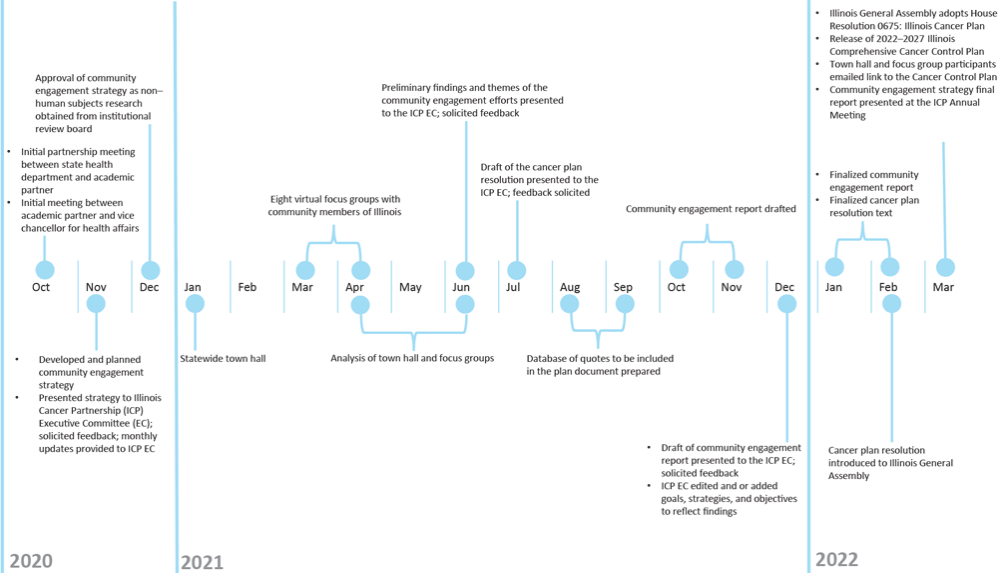
Timeline of activities for creating and implementing a community engagement strategy for the 2022–2027 Illinois Comprehensive Cancer Control Plan.

The process to develop a community engagement strategy for the 2022–2027 Illinois Comprehensive Cancer Control Plan began with a meeting in October 2020 between the state health department partner and the academic partner (Figure 1). The goal of this meeting was to create a partnership that used a health equity lens to engage diverse community members and discuss the resources and assets that each partner was able to provide. Partners identified the following goals, which guided all subsequent activities:

**Table:** 

Month/year	Activity
**2020**	
October	Initial partnership meeting between state health department and academic partner; initial meeting between academic partner and vice chancellor for health affairs
November	Developed and planned community engagement strategy; presented strategy to Illinois Cancer Partnership (ICP) Executive Committee (EC); solicited feedback; monthly updates provided to ICP EC
December	Approval of community engagement strategy as non–human subjects research obtained from institutional review board
**2021**	
January	Statewide town hall
March–April	Eight virtual focus groups with community members of Illinois
April–June	Analysis of town hall and focus groups
June	Preliminary findings and themes of the community engagement efforts presented to the ICP EC; solicited feedback
July	Draft of the cancer plan resolution presented to the ICP EC; feedback solicited
August–September	Database of quotes to be included in the plan document prepared
October–November	Community engagement report drafted
December	Draft of community engagement report presented to the ICP EC; solicited feedback; ICP EC edited and/or added goals, strategies, and objectives to reflect findings
**2022**	
January–February	Finalized community engagement report; finalized cancer plan resolution text
February	Cancer plan resolution introduced to Illinois General Assembly
March	Illinois General Assembly adopts House Resolution 0675: Illinois Cancer Plan; release of 2022–2027 Illinois Comprehensive Cancer Control Plan; town hall and focus group participants emailed link to the Cancer Control Plan; community engagement strategy final report presented at the ICP Annual Meeting

- **Goal 1:** Develop a strategy to engage diverse perspectives in the development process for the 2022–2027 Illinois Comprehensive Cancer Control Plan.
- **Goal 2:** Elicit community feedback on the plan’s goals and objectives, focusing on addressing cancer inequities in Illinois.
- **Goal 3:** Raise awareness of the development of the plan among Illinois legislative and community members, coalition members, and others with a vested interest in addressing cancer needs in Illinois.

**Figure 2:**
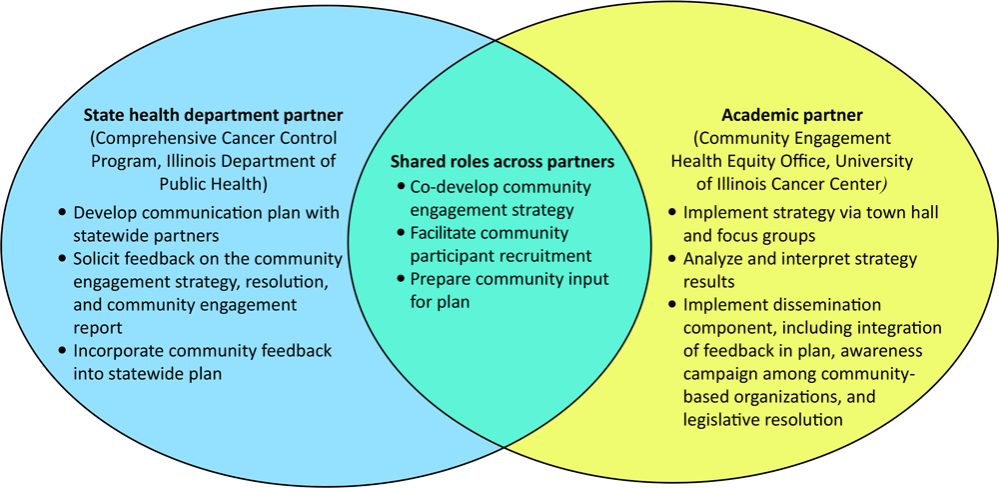
Unique and shared roles and responsibilities of the state health department and academic partners for the community engagement strategy for the 2022–2027 Illinois Comprehensive Cancer Control Plan.

The partners completed a partnership agreement template (Appendix) to establish ground rules for collaboration and determine desired level of collaboration, based on the Collaboration Spectrum Tool (9). The levels of partnership include cooperate, coordinate, collaborate, and integrate. The agreement summarized mutual benefits and described alignments with each partner’s strategic priorities, guidelines for authorship, and partners’ roles and scope of work (Figure 2) (9,10).

### Implementation of the community member engagement strategy

By consensus, partners determined that the community engagement strategy would include a virtual town hall meeting and a series of 8 virtual focus groups. The overall objectives of the town hall and focus groups were to identify 1) cancer-related problems, barriers, and gaps that people in Illinois experience; 2) solutions, facilitating factors, and strengths to address the problems; and 3) funding priorities. The town hall was hosted first, followed by the 8 focus groups that delved deeper into topics about health equity and cancer disparities.

The University of Illinois Institutional Review Board reviewed an application for the determination of human subjects research and granted this project (protocol no. 2020–1552) a formal Determination of Quality Improvement status.

### Recruitment and eligibility

We recruited participants for both the town hall and the focus groups primarily through flyers sent via email to the academic and state health department partner networks throughout the state. In addition, flyers were distributed to the ICP listserv of approximately 600 people, including health practitioners and administrators from city, county, and state health departments, and hospitals and community health centers; representatives of cancer advocacy organizations; cancer survivors and caregivers of cancer patients; and researchers and clinicians, all of whom were encouraged to distribute the flyers through their own networks and social media.

The town hall was held during the day in January 2021 and was open to all interested adults residing in Illinois; online preregistration was required. People who completed the registration process received a follow-up email with a link to the meeting, followed by at least 2 reminder emails. We asked town hall participants to indicate their sex, race and ethnicity, whether they were a cancer survivor or caregiver for a cancer patient, and affiliation (eg, community member, hospital, government agency). We did not ask town hall participants to indicate age, health insurance coverage, preferred language, or residence (rural vs urban).

For the focus groups, held in March and April 2021, we used purposive sampling methods to select participants to ensure broad representation based on race and ethnicity, cancer survivors and caregivers, health insurance status, and residence (urban vs rural). Potential participants completed a registration form, which included questions on demographic characteristics (race and ethnicity, sex, age, preferred language, and rural vs urban residence). Rural or urban residency was based on the person’s perception of place and not a specific classification system. People were not required to participate in the town hall to sign up for focus groups. Several focus groups were offered in the evening or during the weekend to promote participation among those who may not have availability during the week.

### Town hall and focus group procedures

The town hall and focus groups procedures were organized and aligned with CDC’s Community Health Assessment and Group Evaluation (CHANGE) Action Guide (11) and the Center for Community Health and Development at the University of Kansas’ Community Tool Box (12), both of which provide guidance and best practices for engaging with community members to understand and assess health disparities.

The academic partner developed semistructured moderator guides for the town hall and focus groups (Table 1). The moderator guides were based on a model for the analysis of population health and health disparities (13), which incorporates a multilevel lens to understand factors that contribute to health disparities: fundamental causes, the social and physical context, individual demographics and risk factors, and biologic responses and pathways.

**Table 1:** Sample Questions From the Moderator Guide for the Town Hall and Focus Groups, Community Engagement Strategy for the 2022–2027 Illinois Comprehensive Cancer Control Plan^a^

Multilevel factor	Description	Sample questions
Fundamental causes	Includes social conditions and policies (eg, poverty, public policy, culture, norms, discrimination) and the institutional context (eg, health care system; economic, legal, political systems).	• Now, I would like you to think about yourself, a loved one, or someone from your community who was diagnosed with cancer. How easy or hard would it be for this person to get the information to make decisions about their care? • How easy or hard would it be for this person to get good, high-quality treatment? • What would make it easier for this person to get the care they need?
Physical and social context	Includes physical context (eg, pollution, transit access, parks), social context (eg, collective efficacy, social capital, racial and ethnic integration), and social relationships (eg, social networks, social support, civic engagement).	• Health disparities are differences that we see in health and health care between groups. These groups can be based on race, where you live, your income level, gender, sexual orientation, age, or physical abilities, among other things. For example, some groups have worse health outcomes related to cancer and less access to care than others. What do you think are some of the things in your community that contribute to cancer disparities? • What are some of the best ways to improve cancer disparities?
Individual demographic characteristics and risk factors	Includes individual demographic characteristics (eg, age, socioeconomic status, health status) and individual risk behaviors (eg, tobacco use, engagement in health care system).	• Would you tell me about a time when you knew you needed to get a recommended cancer screening, but decided not to do it, or put it off for a period of time? • Now, think about a time when you knew you needed to get a cancer screening and you did. What helped you take that action?
Biologic responses and pathways	Includes biologic responses (eg, stress, hypertension, previous illness) and biologic and genetic pathways (eg, allostatic load, genetic ancestry).	• How, if at all, is cancer talked about in your family? • How has this influenced your use of getting screened for different cancers?

a The moderator guides were informed by a model for analysis of population health and health disparities (13), which articulates multilevel factors that are important to consider when seeking to understand disparate health outcomes: fundamental causes, the social and physical context, individual demographics and risk factors, and biologic responses and pathways.

The town hall, which included a breakout session, was hosted by the academic partner and lasted 90 minutes. It was used as an opportunity to raise awareness of the cancer control plan and recruit focus group participants. The town hall began with introductions from the state health department and academic partners. Participants were randomly assigned to 1 of 5 breakout rooms to delve into specific cancer-related topics, with a facilitator and notetaker from the academic partner in each room. After the breakout sessions, participants returned to the town hall and were invited to complete online registration for a focus group.

The academic partner hosted and facilitated the focus groups in March and April 2021. Of the 8 focus groups, 3 were for the general population and each of the other 5 was tailored for a specified group: rural residents, cancer survivors, young cancer survivors, cancer caregivers, and Spanish speakers. All focus groups were recorded and lasted from 75 to 98 minutes (mean, 83 min). On average, each group had 7 participants (range, 5–10). Participants received a $40-equivalent gift (gift card, electronic code, or digital payment) to acknowledge their time and effort and decrease barriers to participation. Before the town hall and focus groups, the academic partner held 3 one-hour training sessions for facilitators to review the basic principles of conducting qualitative data collection, building participant rapport, asking good questions and probes, and managing group conversations.

### Analysis of data from town hall and focus groups

The town hall was not recorded because of technical problems in using breakout rooms in the Zoom platform, but a notetaker was assigned to the main town hall Zoom room in addition to the notetakers in the breakout rooms. Immediately after the town hall, the facilitators and notetakers reviewed and discussed notes, organized them topically, and listed key themes.

All focus groups were recorded via Zoom. Before analysis, all focus groups were transcribed, checked for accuracy, and de-identified. We used Dedoose version 9.0.18, a web-based qualitative data software application to conduct analysis. Members of the analytic team (L.C., C.H., B.S.) used content analysis procedures and developed a codebook to identify themes and subthemes. The final codebook consisted of 58 codes. Finally, themes were organized according to the model for analysis of population health and health disparities (13), and sample quotes were extracted to illustrate themes. 

At the time of the focus group and town hall, the ICP had already drafted the goals, objectives, and strategies to be included in the 2022–2027 Illinois Comprehensive Cancer Control Plan. The ICP reviewed the major themes identified by the analysis of data from the town hall and focus groups and revised the plan according to this analysis.

### Implementation of the legislative body engagement strategy

The academic partner, in consultation with the ICP and the state health department partner, initiated engagement with the Illinois legislative body by drafting a resolution to raise awareness of a state cancer plan and the development of the 2022–2027 plan. A resolution is a statement of opinion that does not have the force of law. Because of rules and laws about lobbying, the state health department partner was not permitted to engage with the legislative body. The academic partner, in consultation with their institution’s vice chancellor for health affairs office, whose function is health affairs advocacy and government relations, created the initial draft of the resolution. The draft was then shared with the ICP for input, which was incorporated into the final version. Next, the academic partner, in collaboration with the vice chancellor for health affairs office, contacted the American Cancer Society’s director of government affairs for Illinois to leverage their expertise in advocacy and policy work. The academic partner facilitated a meeting between the vice chancellor for health affairs office and the American Cancer Society, wherein it was decided that the next step would be to engage the Illinois Joint Legislative Cancer Caucus to seek support for adoption of the resolution. Finally, a schedule to contact the chair of the Cancer Caucus was set to align with the state’s legislative session calendar.

## Results

### Community member engagement

In total, 112 people participated in the community engagement strategies; 59 (53%) participated in the town hall only, 50 (45%) in the focus groups only, and 3 (3%) participated in both (Table 2). Overall, 102 (91%) participants self-reported their sex as female; 18 (16%) self-reported Hispanic, Spanish, or Latino origin; 26 (23%), Black or African American; 75 (67%), White; and 7 (6%), Asian. Focus groups participants on average were aged 52 (SD, 14; range, 25–88) years. Of 53 focus group participants, 5 (9%) reported a preference for Spanish language, and 14 (26%) were rural residents. Of the 62 town hall participants, 22 (35%) reported an academic affiliation, 10 (16%) reported being from a community-based organization, and 8 (13%) reported being from a hospital or clinical setting.

**Table 2:** Characteristics of Participants in Community Engagement Strategy (N = 112) for the 2022–2027 Illinois Comprehensive Cancer Control Plan

Characteristic	No. (%)
**Participation**	
Participated in town hall only	59 (53)
Participated in focus group only	50 (45)
Participated in both town hall and focus group	3 (3)
**Sex**	
Female	102 (91)
Male	9 (8)
Sex not reported	1 (1)
**Hispanic, Spanish, or Latino origin**	18 (16)
**Race**	
American Indian or Alaska Native	1 (1)
Asian	7 (6)
Black or African American	26 (23)
White	75 (67)
Race not reported	3 (3)
**Cancer survivor**	40 (36)
**Caregiver for a cancer patient (current or past)**	31 (28)
**Affiliation^a^**	
Community member	5 (8)
Academic affiliate	22 (35)
Community-based organization	10 (16)
Hospital or clinical setting	8 (13)
Government agency or health department	5 (8)
**Age, y^b^^,^ c**	
<40	12 (23)
40–59	25 (47)
>60	16 (30)
**Current health insurance coverage^b^^,^ d**	
Private	33 (62)
Public	14 (26)
Other source of coverage	3 (6)
Uninsured, no coverage	3 (6)
**Preferred language for focus group^b^**	
English	48 (91)
Spanish	5 (9)
**Current rural residence^b^**	14 (26)

a Question asked only of town hall participants (n = 62); they were asked to select all that apply.

b Question asked only of focus group participants (n = 53).

c Mean (SD) [range] = 52 (14) [25–88] years.

d Public insurance includes Medicare, Medicaid, or coverage through the Affordable Care Act.

### Major themes from town hall and focus group analysis

Participants in the town hall and focus groups described factors that contribute to cancer disparities among people in Illinois.

The town hall participants discussed the importance of understanding and addressing health disparities broadly and specifically to cancer throughout the CCC plan. One town hall attendee stated, “Cancer affects everyone but not everyone equally.” Determinants of health, such as access to food, safe physical activity, transportation, health insurance coverage, access to health care providers (including specialists), treatment options (including second opinions and clinical trials), and knowledge about health, health care systems, and available resources were discussed extensively, especially as they pertained to racial and ethnic groups and immigrant status in Illinois. Participants also discussed how access to transportation and cancer care resources (ie, patient navigators, specialty care), the digital divide, and exposure to environmental hazards depend on where one lives in Illinois. They recommended that the plan include education and awareness of multiple cancer types; highlight the importance of early detection, patient navigation, and collaboration with health systems and organizations; and ensure that goals and objectives are realistic and attainable. Finally, the COVID-19 pandemic was a major topic of discussion, especially concerns about exposure among cancer patients and survivors and disruption of the health care system and cancer care.

Key themes from the focus groups largely mirrored those from the town hall (Table 3). Overall, the lack of a comprehensive health insurance system in Illinois and discrimination based on race and ethnicity and immigration status were identified as being the primary policy and social conditions that contributed to cancer disparities across the cancer continuum. Concerning the institutional context, lack of access to quality systems and services was a recurring theme. Concerning the physical context, participants discussed the importance of where one lives and how place relates to community and individual health outcomes. Specifically, participants discussed environmental hazards, internet access and the digital divide, transportation, and food insecurity as subthemes. Access to health care and transportation challenges were noted among both rural and urban residents, although we found nuanced differences. For example, rural residents talked more about a lack of medical facilities overall, and urban residents talked more about access in terms of quality of care. Furthermore, urban residents noted access to supports and resources that are available to people living in an urban center, whereas rural residents often discussed a lack of resources to address needs across the cancer continuum (ie, education and prevention resources, care navigation services, innovative diagnostic and treatment care, and survivor peer and social support).

**Table 3:** Sample Quotes From Focus Group Participants About Multilevel Factors That Contribute to Cancer Disparities Among People in Illinois, Community Engagement Strategy for the 2022–2027 Illinois Comprehensive Cancer Control Plan

Multilevel factor	Sample quote
**Fundamental causes of cancer disparities**	
• Access to quality care, clinical trials, patient navigation services • Social conditions and policies, including lack of comprehensive health insurance system • Institutional context, including lack of access to quality systems and services • Discrimination	[Access to a research institution] is literally a lifeline. You have access to clinical trials . . . and the response time is phenomenal if you’re in a location that has that kind of infrastructure. But most . . . in this country do not live near a major research hospital. And I do not expect that we can be successful at treating cancer early, or even getting people treatment that they need, without the access. Access is everything. [African American cancer survivor from Cook County, aged 60 years] My insurance is through the Affordable Care Act. When Illinois extended Medicaid to cover low-income individuals, I qualified. And I find having that as my insurance affects who I can see. . . . I feel that the quality of health care I’m getting . . . because of my insurance is less. It isn’t as good. [Non-Hispanic White cancer survivor from central Illinois, aged 57 years] And I’ve heard from friends, in particular, friends who are not White, who do not feel like doctors trust or actually listen to them and validate what they’re experiencing. [Non-Hispanic White cancer survivor from Cook County, aged 34 years]
**Physical context**	
• Location (rural vs urban) • Environmental hazards • Digital divide and telehealth • Transportation • Food insecurity	So, if you’re in a hard-to-reach region, why should you get third-tier treatment? It’s an unequal distribution of medical care in the state and it has been for a very, very long time. [Non-Hispanic White cancer survivor from rural central Illinois, aged 55 years] I think if one lives in an urban area, your air quality probably isn’t very good. So, that probably has a lot to do with cancer diagnoses. [Non-Hispanic White cancer survivor from Cook County, aged 58 years] I mean, not everyone has access to a vehicle. . . . Quite often, the medical profession doesn’t consider that. . . . And in a way, it’s like blaming the victim because I don't have access to what I need to get there. . . . Access for me is difficult. And somehow, they never seem to ask those questions. You know, what can we do to help you get here? Do you need a ride? Something like that. None of that becomes a conversation. [African American cancer survivor and caregiver from central Illinois, aged 49 years]
**Social context**	
• Fear of cancer in communities • Community characteristics • Residential segregation • Social networks and norms • Patient–provider relationships	I think part of it is the fear of the expense of medical care, not understanding it — having insurance or not, understanding insurance, and that financial fear. [Asian cancer survivor and caregiver from Cook County, aged 63 years] Having a conversation with the doctor may not be as understandable, and people don’t know how to continue to say, “I don’t understand” or “tell me in a different way.” So, it’s also a point of literacy and understanding. So, the doctor went to medical school and he or she is an expert. But if they can’t deliver that message and that information in a way that’s understandable, then they haven’t done a good job. And so, I may sit in the office, I may get lots of information which is good pertinent information. But if I don’t understand it, I don’t have anything. [African American cancer survivor and caregiver from central Illinois, aged 49 years]
**Individual demographics, risk factors, and biologic responses and pathways**	
• Insurance status • Immigration status	You know, I’m blessed to have a husband, and I’ve told him many times that were it not for our insurance coverage, I don’t know where I’d be. Ovarian cancer is a very expensive treatment. CT scans are 12,000 dollars sometimes. I just don’t know how people could do it if they were not covered. I really don’t. [Latina cancer survivor and caregiver from Cook County, aged 48 years] There’s a lot of people who have the thought that [name of public hospital] isn’t good because that’s where all of the immigrants go, but . . . people who don’t have resources to go to another hospital go there, and that’s why they take a long time. Personally, I can say that years ago it was like that. . . . They gave me an ultrasound [appointment] in 6 months. When I got to 6 months, I didn’t have the pain anymore. [Latina community member from Cook County, aged 48 years] Once we found out there was a genetic mutation in the family — so now, one of my cousins who’s younger than me, she actually got screened for it and so she’s talking to a specialist to see what her options are so that she has more of a choice with it. [Non-Hispanic White cancer survivor from rural southern Illinois, aged 36 years]

Focus group participants also discussed the importance of the social context and how factors such as community poverty, residential segregation, and inadequate social networks contribute to cancer disparities in the state. Rural residents noted their large aging populations and discussed age-related challenges. Participants discussed the effect of individual-level risk factors and health behaviors on cancer disparities, but when they mentioned these, they typically connected these factors with the social and physical community contexts that shape behavior, such as access to resources, safety, and engagement in physical activity.

### Recommendations and funding priorities to improve health across the cancer continuum

Participants in the town hall and focus groups recommended policy and systems, clinical, community, and individual-level strategies and funding priorities to address cancer disparities in Illinois. The recommendations spanned the entire cancer care continuum, from prevention, screening, diagnosis, and treatment to survivorship and palliative care. The primary policy concern was ensuring that all who need cancer care are able to receive it, regardless of cost and ability to pay. Clinical-level recommendations to address cancer disparities included access to patient navigation, improved patient–provider communication, and training for health care providers. Community-level recommendations included increased access to community navigators, ensuring that transportation needs are met for both rural and urban communities, and addressing food insecurity by establishing food depositories throughout the state. Finally, individual-level recommendations included the need to increase awareness and education opportunities about cancer.

Town hall and focus group participants shared their ideas about how funds should be prioritized in Illinois to address cancer. First, they noted that community organizations, especially those addressing cancer disparities and working collaboratively, should be prioritized for funding. They also suggested prioritizing funding for cancer prevention and research; programs that provide social, emotional, and educational support; and patient navigation services. Finally, participants mentioned that funding needed to be spread out across different types of cancers.

The complete report on the results of the town hall and focus groups can be found in the 2022–2027 Illinois Comprehensive Cancer Control Plan (14). The report incorporated participant quotes to support specific goals, objectives, and strategies. In addition, 8 infographics were created to support dissemination (15).

### Legislative members engagement strategy

The chair of the Illinois Joint Legislative Cancer Caucus agreed to be the primary sponsor of Illinois House Resolution 0675, the 2022–2027 Illinois Comprehensive Cancer Control Plan (16), and garnered cosponsorship from other legislators. The resolution, adopted on March 15, 2022, approximately 1 month after it was filed, urged all legislators to support and promote the plan to address 3 priority areas (prevention; early detection and screening; and diagnosis, treatment, and survivorship) by engaging, educating, and empowering constituents through community engagement. The resolution discusses social determinants of health and recognizes the need to address cancer health equity and eliminate health disparities by providing a framework for strategies and interventions that address structural and systemic barriers.

## Discussion

We implemented a robust community engagement strategy through a successful state health department–academic partnership. Our work informed the development of the 2022–2027 Illinois Comprehensive Cancer Control Plan and helped raise awareness of the plan among Illinois legislators. This model of community engagement can be replicated by other coalitions that are developing state cancer plans or other similar documents. Many states are already using similar approaches to prepare their plans. For example, Indiana (17), Nebraska (18), and Tennessee (19) used town halls and focus groups to understand community priorities What is unique about the community engagement approach is that it is explicitly centered in health equity theory, which promotes understanding cancer concerns at multiple levels. Relatedly, many states have described using collaborative approaches that involve multiple partners. However, many descriptions lack details about the various roles and responsibilities involved in planning. Our work described and delineated unique and shared roles and responsibilities of academic and state health department partners.

Using community engagement approaches ensured that the Illinois plan reflects the voices of people affected by cancer in Illinois and the diverse needs and assets in the state. Our approach was guided by public health models of engagement and theoretical models of social determinants of health (11–13). This approach emphasized understanding and addressing not only the role of individual-level risk factors and behaviors in cancer health disparities but also the role of fundamental causes and physical and social contexts. This approach may also be considered for creating strategic plans to address other chronic conditions.

### Limitations

Our community engagement strategy has several limitations. First, we did not have a transcription of the town hall meeting, so we were unable to review verbatim comments. However, the academic partner had notetakers for the town hall and for each breakout room. Second, recruitment focused on ensuring representation of participants by rural and urban residence, health insurance status, and race and ethnicity. Thus, the perspective of some populations (eg, men, people with gender identities other than male or female) may be limited. Coalitions could consider recruitment strategies that take this limitation into account. Third, we used self-reported information on rural residency, and a participant’s perception of rural residency may not match an objective measure. However, we wanted to acknowledge the validity of lived experiences. Finally, because of the timing of our work and the COVID-19 pandemic, we were unable to have in-person events. Although virtual focus groups have some advantages, such as mitigating travel challenges and reaching diverse populations, virtual modalities are less likely to reach people without access to or the capacity to use technology (20,21).

### Conclusion

We recommend that the ICP and other coalitions working on cancer plans develop strategies to include community members in the development of plan goals, objectives, and strategies. Although the 2022–2027 Illinois Comprehensive Cancer Control Plan considered community feedback before these elements were finalized, soliciting this input at the onset would have increased community engagement and participation.

Our community engagement strategy reflects a process through which the expertise and voices of community members can be documented and reflected in state CCC plans. We highlighted a mechanism through which plans can be brought to the attention of legislators. Other coalitions working on their state’s plans could consider replicating some or all of our strategy. Ultimately, plans should reflect principles of health equity and prioritize the elimination of cancer disparities.

## Appendix Text of Community Engagement and Health Equity Office Community Partnership Agreement

This agreement is between The Community Engagement and Health Equity (CEHE) Office, as a part of the University of Illinois Cancer Center and [community organization], each wishing to establish a working relationship to support [Project Work].

[Community organization/partner] and CEHE wish to enable a [level of partnership] partnership and exchange in the Project Work by working together agreeing to the below:

1. Background. [Provide a brief background of the relationship between the partners.]

- Include each organization’s mission
- List who the primary contacts are:
- CEHE primary contact/principal investigator/lead staff
- Community organization/partner

2. Goals of partnership. This agreement reflects the genuine intentions to form a working relationship. The purpose of this agreement is to advance the ideas and activities to meet the following goals:

- Goal 1:
- Goal 2:
- Goal 3:

3. Summary of mutual benefit.

- Based on Give/Get model

What contributions and benefits can the community organization and the community engagement cancer center team share to [work together on (ie, develop or create a plan)] to reach [goals (ie, increase cervical cancer awareness)]

What community can give? What community can get?

What CEHE can give? What CEHE can get?

- What is the alignment with CEHE’s strategic priorities?
- What is the alignment with community organization’s strategic priorities?

4. Authorship and acknowledgments.

- List and order of authorship must be discussed before the development of any manuscripts as a result of the partnership.
- Products as a result of this partnership should include an acknowledgment of the Community Engagement and Health Equity Office at the University of Illinois Cancer Center. Products and publications include research and technical papers, preprints, conference and academic presentations, theses and dissertations, journals and books, oral histories, video and audio recordings of speeches and events, photographs, and key project documents.
- Both partners should be branded together with their respective logos on any promotion or dissemination.
- Partners agree that ownership of intellectual property rights generated as a result of the activities under this agreement will follow inventorship rule and remain the property of the partner introducing and/or disclosing the same to the other partner.

5. Description of engagement deliverables and timeline (scope of work).

Include activities/deliverables, persons responsible for conducting each activity and timeline for completion

6. Mutual agreement time period.

- This agreement will remain in effect for one (1) year from the date of the last signature. Either partner may terminate this agreement by informing the other partner with an electronic or written form of communication.

7. Evaluation.

- The partners will convene twice a year to focus discuss partnership status, communication and outcomes

Signed for and on behalf of:

By: [Community organization/partner] [Title]

Date:

By: [CEHE] [CEHE authorized official] [Title]

Date:

AGREEMENT is effective for one year after last signature has been provided; date: ________________
